# Age at First Experience of Gender Dysphoria Among Transgender Adults Seeking Gender-Affirming Surgery

**DOI:** 10.1001/jamanetworkopen.2020.1236

**Published:** 2020-03-16

**Authors:** Michael Zaliznyak, Catherine Bresee, Maurice M. Garcia

**Affiliations:** 1 Division of Urology, Cedars-Sinai Medical Center, Los Angeles, California; 2 Biostatistics and Bioinformatics Core, Cedars-Sinai Samuel Oschin Comprehensive Cancer Center, Los Angeles, California; 3 Department of Urology, University of California, San Francisco; 4 Department of Anatomy, University of California, San Francisco; 5 Cedars-Sinai Transgender Surgery and Health Program, Cedars-Sinai Medical Center, Los Angeles, California

## Abstract

This cross-sectional study examines the age at which US transgender adults first experienced gender dysphoria and the number of life-years that transgender adults lived with untreated gender dysphoria and its comorbidities before seeking gender-affirming surgery.

## Introduction

To our knowledge, the mean earliest age at which adult transgender women (TW) and transgender men (TM) first experienced gender dysphoria (GD) has not been quantified and reported among a US population of adults seeking genital gender-affirming surgery (GAS). Because gender is an innate part of an individual’s identity, we hypothesized that untreated GD would be part of an individual’s earliest memories. Our aim was to identify the age at which patients first experienced GD and the number of life-years that a diverse US sample of transgender adult presurgical patients spent living with untreated GD and its comorbidities. This information is important because it can help guide health care practitioners to as to when and how to best focus outreach and care to patients not yet identified as transgender.

## Methods

The Cedars-Sinai Medical Center institutional review board reviewed and approved this cross-sectional study. This study was conducted in accordance with all relevant guidelines and procedures of Cedars-Sinai Medical Center. A waiver of informed consent was granted by the institutional review board for the collection and reporting of the data because they were deidentified.

All study data were obtained at a single study site (Cedars-Sinai Medical Center). All participants received a diagnosis of GD from a mental health specialist in accordance with *Diagnostic and Statistical Manual of Mental Disorders*, Fifth Edition, criteria. All patients were presenting for consultation for GAS and already met the criteria for surgery. During consultation for genital GAS, all consecutive TW and TM were asked to report how old they were at their earliest general (non–gender-related) memories and how old they were at their first experience of GD.

Pearson correlation coefficients were computed to test the association between variables. Two-sample *t *tests were used to test for differences in mean ages. Data are presented as mean (SD). Differences were considered statistically significant where 2-tailed *P* < .05. Data were analyzed using SAS statistical software version 9.4 (SAS Institute). Data analysis was performed in April 2019.

## Results

Data from 155 TW (mean [SD] age, 41.3 [16.3] years) and 55 TM (mean age 35.4 [10.8] years) were collected (Table 1). Approximately one-half (52%) of all TM and TW were white and non-Hispanic (110 patients), and nearly one-half (101 patients [48%]) had an active or recent history of anxiety or depression. Among TW, 7% (11 patients) were HIV positive. For TM, the mean (SD) age of earliest general memory and age at first experience of GD were 4.7 (2.3) and 6.2 (3.1) years, respectively; for TW, these ages were 4.5 (2.0) and 6.7 (3.6) years, respectively (Table 2). Most of the patients (112 TW [73%] and 41 TM [78%]) first experienced GD by the age of 7 years. TM and TW lived for a mean (SD) of 22.9 (12.6) and 27.1 (16.4) years, respectively, with untreated GD before commencing gender transition (nonsurgical). Birth sex, age at commencement of transition, sexual orientation, and age at presentation for surgery were not associated with age at earliest memory of GD.

**Table 1.** Demographic, Social, and Clinical Characteristics of Transgender Patients

Characteristic	Patients, No. (%)	
	Men (n = 55)	Women (n = 155)
Age, mean (SD), y	35.4 (10.8)	41.3 (16.3)
Age range at consultation for surgery, y		
16-19	0	8 (5)
20-29	19 (35)	38 (25)
30-39	13 (24)	29 (19)
40-49	15 (27)	22 (14)
50-59	6 (11)	30 (19)
≥60	2 (4)	28 (18)
Race/ethnicity		
White (non-Hispanic)	26 (47)	84 (54)
Hispanic	10 (18)	30 (19)
Black or African American	7 (13)	12 (8)
Asian or Pacific Islander	4 (7)	19 (12)
Not disclosed	8 (15)	10 (6)
Gender of sexual partners		
Patients, No.	52	153
Men	6 (12)	71 (46)
Women	38 (73)	50 (33)
Both	8 (15)	32 (21)
Mental health comorbidities		
Depression	7 (13)	17 (11)
Anxiety	5 (9)	10 (6)
Both	17 (31)	45 (29)
Neither	26 (47)	83 (54)
Reported history of suicide attempt?		
Patients, No.	48	149
Yes	10 (21)	45 (30)
No	38 (79)	104 (70)
Suicide attempts, mean (SD) [range], No.	1.9 (1.3) [1-5]	1.8 (1.7) [1-10]
Reported current feelings of suicide ideation?		
Patients, No.	48	149
Yes	1 (2)	12 (8)
No	47 (98)	137 (92)
Medical HIV comorbidities		
HIV positive	0	11 (7)
HIV negative	55 (100)	144 (93)

**Table 2.** Earliest Age of First Memory of GD Among Transgender Patients

Variable	Patients, No. (%)	
	Men	Women
Age at time of earliest memory, mean (SD), y	4.7 (2.3)	4.5 (2.0)
Patients, No.	30	101
Age at first GD, mean (SD), y	6.2 (3.1)	6.7 (3.6)
Patients, No.	52	155
Age range at first memory of GD, y		
Patients, No.	52	155
2-4	16 (30)	38 (25)
5-7	25 (48)	74 (48)
8-10	4 (8)	19 (12)
11-13	5 (10)	17 (11)
14-24	2 (4)	7 (5)
Rank their earliest memory of GD as among their earliest life memories		
Patients, No.	30	101
Yes	24 (80)	82 (81)
No	6 (20)	19 (19)
Duration patients lived with GD before commencing gender transition (nonsurgical), y		
Patients, No.	50	150
<10	7 (14)	20 (13)
10-19	17 (34)	40 (27)
20-29	13 (26)	33 (22)
30-39	6 (12)	23 (15)
40-49	5 (10)	16 (11)
≥50	2 (4)	18 (12)
Mean (SD) [range]	22.9 (12.6) [3-58]	27.1 (16.4) [1-68]

Abbreviation: GD, gender dysphoria.

## Discussion

To our knowledge, this study is the first to quantify the earliest age at which GD was experienced in a US patient population seeking genital GAS. We found that nearly all TM and TW first experienced GD by age 7 years (gender identity typically becomes constant at ages 5-7 years),^1^ which is only 1.5 and 2.2 years later than each cohort’s first life memories (which typically occur at ages 3-4 years).^2^ Our findings are consistent with prior research^3^ that found that GD generally develops early in life.

Another important finding from our study is the number of years that transgender adults live with untreated GD before commencing treatment (social transition and/or hormonal therapy): 22.9 years for TM and 27.1 years for TW. When we consider that life-years spent with any untreated condition are associated with morbidity and decreased quality of life, then the high number of life-years spent living with GD and its associated morbidities is striking.^4,5^ This large cumulative burden strongly supports the argument for early GD care, counseling, and education about transition-related options, both for patients and for their parents or guardians.^6^ Gender transition–related care has been shown to substantially improve quality of life and reduce health care costs.^3,4,5,6^

This study has some limitations. The participants included only patients presenting for genital GAS and, thus, may not be representative of transgender people who do not seek GAS. Patients were not queried about the severity of their GD and mental health during their childhood, when they may have experienced a different degree of distress and/or other consequences.

## Conclusions

Our findings suggest that GD typically manifests very early in childhood and can persist for many years until gender transition. As such, GD and its associated adverse health effects are conditions of childhood as much as adolescence or adulthood. Diagnosis and early management during childhood and adolescence can decrease morbidity and improve quality of life and overall survival.

## References

[1] Ruble DN, Taylor LJ, Cyphers L, Greulich FK, Lurye LE, Shrout PE The role of gender constancy in early gender development. Child Dev. 2007;78(4):-. doi:10.1111/j.1467-8624.2007.01056.x 17650129

[2] Tustin K, Hayne H Defining the boundary: age-related changes in childhood amnesia. Dev Psychol. 2010;46(5):1049-1061. doi:10.1037/a0020105 20822222

[3] Heylens G, Elaut E, Kreukels BP, Psychiatric characteristics in transsexual individuals: multicentre study in four European countries. Br J Psychiatry. 2014;204(2):151-156. doi:10.1192/bjp.bp.112.121954 23869030

[4] Costa R, Colizzi M The effect of cross-sex hormonal treatment on gender dysphoria individuals’ mental health: a systematic review. Neuropsychiatr Dis Treat. 2016;12:1953-1966. doi:10.2147/NDT.S95310 27536118 PMC4977075

[5] Padula WV, Heru S, Campbell JD Societal implications of health insurance coverage for medically necessary services in the U.S. transgender population: a cost-effectiveness analysis. J Gen Intern Med. 2016;31(4):394-401. doi:10.1007/s11606-015-3529-6 26481647 PMC4803686

[6] Coleman E, Bockting W, Botzer M, Standards of care for the health of transsexual, transgender, and gender-nonconforming people, version 7. Int J Transgenderism. 2012;13(4):165-232. doi:10.1080/15532739.2011.700873

